# Pre‐hospital transfusion of red blood cells. Part 2: A systematic review of treatment effects on outcomes

**DOI:** 10.1111/tme.12659

**Published:** 2020-01-05

**Authors:** Elisabeth C. van Turenhout, Sebastiaan M. Bossers, Stephan A. Loer, Georgios F. Giannakopoulos, Lothar A. Schwarte, Patrick Schober

**Affiliations:** ^1^ Department of Anaesthesiology, Amsterdam UMC Vrije Universiteit Amsterdam Amsterdam The Netherlands; ^2^ Department of Trauma Surgery, Amsterdam UMC Vrije Universiteit Amsterdam Amsterdam The Netherlands; ^3^ Helicopter Emergency Medical Service “Lifeliner 1”, Amsterdam UMC Vrije Universiteit Amsterdam Amsterdam The Netherlands

**Keywords:** damage control resuscitation, emergency medical service, major haemorrhage, mortality, outcome, prehospital transfusion, red blood cells

## Abstract

The primary aim of this systematic review is to describe the effects of prehospital transfusion of red blood cells (PHTRBC) on patient outcomes. Damage control resuscitation attempts to prevent death through haemorrhage in trauma patients. In this context, transfusion of red blood cells is increasingly used by emergency medical services (EMS). However, evidence on the effects on outcomes is scarce. PubMed and Web of Science were searched through January 2019; 55 articles were included. No randomised controlled studies were identified. While several observational studies suggest an increased survival after PHTRBC, consistent evidence for beneficial effects of PHTRBC on survival was not found. PHTRBC appears to improve haemodynamic parameters, but there is no evidence that shock on arrival to hospital is averted, nor of an association with trauma induced coagulopathy or with length of stay in hospitals or intensive care units. In conclusion, PHTRBC is increasingly used by EMS, but there is no strong evidence for effects of PHTRBC on mortality. Further research with study designs that allow causal inferences is required for more conclusive evidence. The combination of PHTRBC with plasma, as well as the use of individualised transfusion criteria, may potentially show more benefits and should be thoroughly investigated in the future. The review was registered at Prospero (CRD42018084658).

## 1 INTRODUCTION

Haemorrhage is a potentially preventable cause of death after major trauma.^1^, ^2^, ^3^ Topical treatment is not always sufficient to control haemorrhage, since it is often non‐compressible.^3^, ^4^, ^5^ The lethal triad of acidosis, hypothermia and coagulopathy is related to haemorrhagic shock, especially when blood loss is treated with liberal crystalloid fluid replacement.^6^ In damage control resuscitation (DCR), blood products are preferred over crystalloids as fluid replacement, while a degree of hypotension is accepted until haemorrhage control has been achieved.^6^, ^7^, ^8^, ^9^, ^10^, ^11^

Through transfusion of red blood cells (RBC), the infusion of large volumes of crystalloids may be avoided, as RBC provide a more effective volume expansion. Haemostasis and thrombosis are promoted^12^ and oxygen carrying capacity restored.^13^

In an effort to decrease mortality through haemorrhage after major trauma, prehospital transfusion of red blood cells (PHTRBC) is increasingly performed. Military medical teams have been transfusing blood products prior to arrival at a surgical unit for years.^14^, ^15^ This may partially explain survival differences between civilian casualties who require massive transfusion (60%) and military casualties (93%).^16^ More recently, civilian emergency medical services (EMS) have also started to carry blood to the scene and transfuse their patients in the prehospital setting.^17^, ^18^

In part 1 of this series, we described the availability and frequency of PHTRBC around the world, and demonstrated that varying transfusion criteria are being used.^19^ However, to date, little is known concerning the effects on patient outcomes. We have therefore conducted a systematic review with the aim to evaluate the effect of PHTRBC in patients treated by EMS on multiple outcomes including mortality, haemodynamic parameters, and the need for further in‐hospital transfusions.

## 2 METHODS

The review was registered at Prospero (website: https://www.crd.york.ac.uk/prospero, identification number: CRD42018084658). This systematic review was conducted in accordance with PRISMA (Preferred Reporting Items for Systematic Reviews and Meta‐Analyses) guidelines.^20^

### 2.1 *Information sources, search strategy and study selection*

PubMed and Web of Science were searched through January 2019. The search strategy and the process of selection of studies were described previously.^19^ For the purpose of this systematic review, only the manuscripts reporting outcome measures after PHTRBC (data on the hemodynamic state, coagulopathy, 24 hours RBC transfusion requirement, length of stay in hospital/intensive care unit [ICU], mortality, or occurrence of complications) were included. For a comprehensive overview of studies reporting outcomes after PHTRBC, controlled studies as well as observational studies were considered for this systematic review.

### 2.2 *Data extraction*

A standardised data extraction sheet was developed, and after testing this on the first 20 articles, it was refined. The setting and type of transport the EMS used (civilian or military, scene or interfacility) and patient characteristics were extracted. Data regarding the effects of prehospital transfusion were collected, including haemodynamic data, coagulopathy, 24 hours RBC transfusion requirement, length of stay in hospital/ICU, mortality and occurrence of complications. Descriptions of problems that arose during PHTRBC are summarised in the text.

Bias was assessed using the Newcastle‐Ottawa Scale.^21^

### 2.3 *Data synthesis*

A priori, we had planned a random‐effects meta‐analysis of the available evidence. However, no controlled studies were identified, and the observational studies carry a risk of residual confounding even if matching or regression‐based techniques were used to control for confounding. Moreover, a high heterogeneity among the studies precluded meaningful pooling of data: Civilian and military studies are not comparable due to fundamental differences in patients, mechanisms of injury and medical facilities. Also within these two groups, differences between patient populations and patient selection, differences between healthcare systems and EMS systems (eg, logistics, staff, equipment, treatment options, response and transport times to definitive care), differences in the type of blood products used (RBC only or a combination of blood products), differences in transfusion criteria, as well as differences in outcome measures are too great to allow a meaningful combined analysis. A meta‐analysis was therefore not performed.

## 3 RESULTS

### 3.1 *Selection of articles*

**Figure 1:**
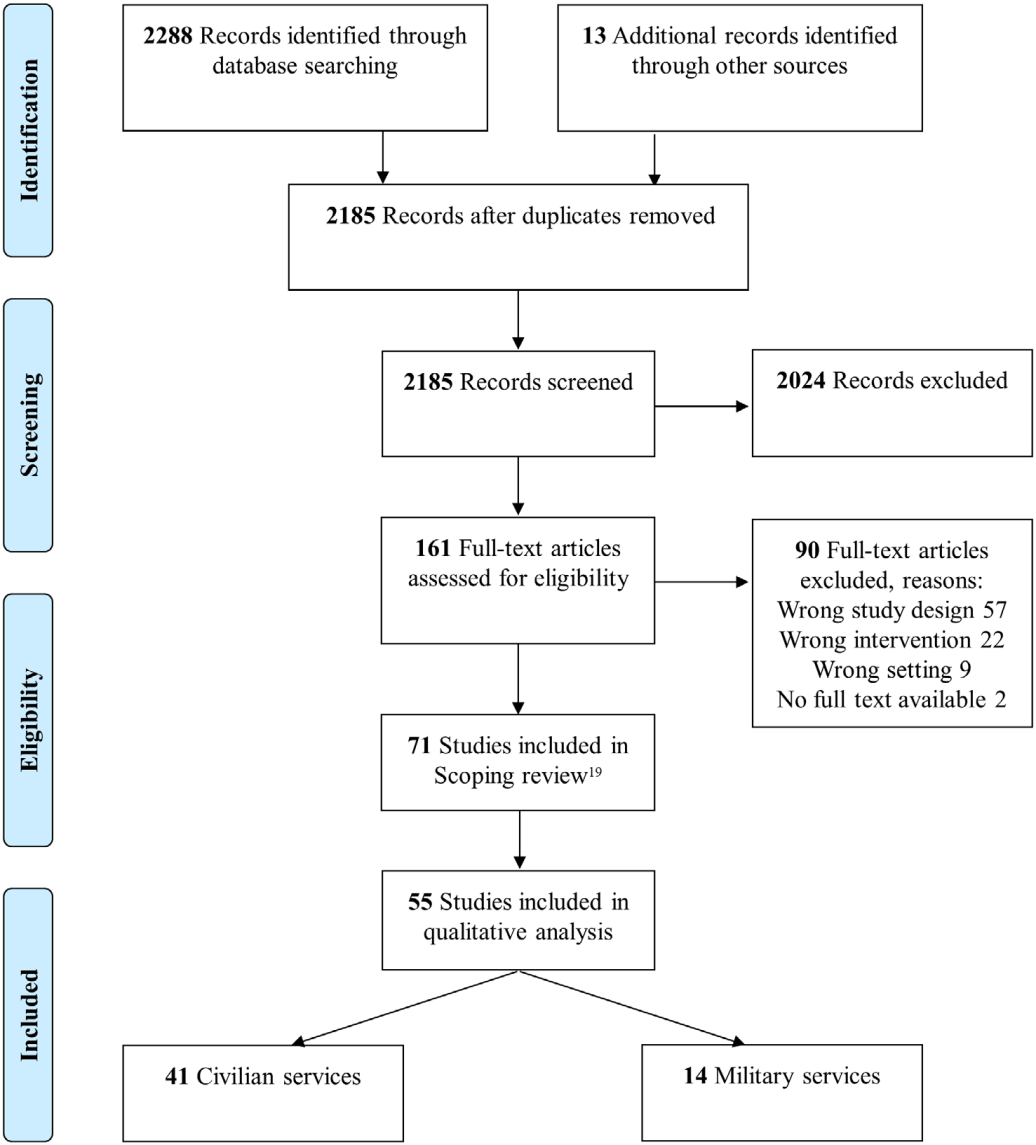
PRISMA flow diagram

The search in PubMed and Web of Science yielded 2172 hits after removal of duplicates. In our scoping review, 71 articles were included.^19^ In total, 55 of these studies reported one or more outcome measure, and were included in this review (Figure 1).

Forty‐one of these studies discussed PHTRBC in civilian medical services, 14 of which allowed comparisons of PHTRBC with a control population. Notably, four articles primarily dealt with a different study topic, but were included as they additionally provided valuable information regarding PHTRBC^22^, ^23^, ^24^, ^25^ (Table 1).

**Table 1:** Overview of studies

First author (y)	Region	Study period	Primary goal	Study group	Control group	Control for confounding	patients transfused (*n*)^a^	Type of transport (%trauma)	Mechanism of injury	ISS
**Civilian services**										
Prospective comparative studies										
Henriksen H (2016)^26^	Texas USA	2012‐2013	To investigate the association between PHTRBC and PHT‐plasma and hemostatic function	Receivers of PHTRBC and/or PHT‐plasma	Receivers of in‐hospital transfusion	Adjusted data	75^b^	Scene (100%)	PHT: Blunt: 55% Penetrating: 45% Control: Blunt: 75% Penetrating: 25% *P* = .002	PHT: 29 (17‐41) Control: 26 (17‐34) *P* = .106
Holcomb J (2017)^27^	USA (9 trauma centers)	2015	To study the effect of PHTRBC and/or PHT‐plasma on in‐hospital mortality	Severely injured receivers of PHTRBC and/or plasma	No prehospital blood products	Propensity score	142^b^	Scene (100%)	PHT: Blunt: 79.1% Penetrating: 20.9% Matched control: Blunt: 72.7% Penetrating: 27.3%	PHT: 24 (10‐34) Control: 22 (10‐34)
Retrospective comparative studies										
Brown JB‐a (2015)^28^	USA (9 institutions)	2003‐2010	To characterise the association of pre‐trauma center RBC with mortality and TIC in severely injured patients with blunt trauma	Receivers of pre‐trauma center RBC	No prehospital transfusion	Propensity score	50	Scene + interfacility (100%)	Blunt: 100% Penetrating: 0% (per exclusion)	PHTRBC: 34 (18‐43) Control: 30 (23‐43) *P* = .81
Brown JB‐b (2015)^29^	Pennsylvania USA	2007‐2012	To evaluate the association of pre‐trauma center RBC with outcomes	Receivers of pre‐trauma center RBC	No prehospital transfusion	Propensity score	240 matched (71 scene)	Scene+ interfacility (100%)	PHTRBC: Blunt: 191(80%) Penetrating: 49(20%) Matched Controls: Blunt: 395(82%) Penetrating: 85(18%)	PHTRBC: 18 (10‐29) Matched Control: 17 (9‐27) *P* = .05
Griggs JE (2018)^30^	Kent Surrey & Sussex UK	2010‐2015	To compare mortality for patients with suspected traumatic haemorrhage receiving PHTRBC compared to crystalloid	Code Red patients receiving PHTRBC	Code Red patients receiving crystalloids	Adjusted data	92	Scene (100%)	PHTRBC: Blunt: 95% Penetrating: 5% MVC 68% Fall 9% Control: Blunt: 99% Penetrating: 1% MVC: 58% Fall: 9%	Mean (SD) PHTRBC:32 (12) Control: 21 (14) *P* = .67
Holcomb JB‐b (2015)^18^	Texas USA	2011‐2013	To evaluate effect of PHTRBC and/or PHT‐plasma on survival and blood product use	Receivers of PHTRBC and/or PHT‐plasma	Receivers of in‐hospital transfusion	adjusted data	137^b^	Scene (100%)	PHT: Blunt: 77% Penetrating: 23% Control: Blunt 83% Penetrating 17% *P* = .447	PHT: 22 (12‐29) Control: 22 (11‐33) *P* = .998
Kim BD (2012)^22^	Minnesota USA	2009‐2011	The analyse the effect of PHT‐plasma on coagulopathy	Receivers of PHT‐plasma + PHTRBC	Receivers of PHTRBC only	no	59 (of whom 50 RBC only)	Scene+ interfacility (100%)	Plasma: Blunt: 67% Penetrating: 33% PHTRBC only: Blunt: 82% Penetrating 18% *P* = .317	Plasma: 27 PHTRBC: 23 *P* = .918
Miller B (2016)^31^	Tennessee USA	2007‐2013	To examine the impact of PHTRBC on mortality	Receivers of PHTRBC	No prehospital transfusion	Propensity score	231 (195 matched)	Scene (100%)	PHTRBC: Blunt: 78% Penetrating: 22% Matched control: Blunt: 90% Penetrating: 10% *P* < .001	PHTRBC: 34 (22‐43) Matched control: 32 (22‐43) *P* = .903
Parker ME (2017)^32^	Minnesota USA	2010‐2014	To examine PHT of plasma and/or RBC on outcomes in exsanguinating GI bleeding	Receivers of PHTRBC and/or PHT‐plasma with acute GI bleeding	Vs GI‐bleed patients without transfusion	No	112^b^	Interfacility (0%)	n/a	n/a
Peters J (2017)^33^	Nijmegen Rotterdam The Netherlands	2007‐2015	To establish the efficacy and safety of the PHTRBC by HEMS	Receivers of PHTRBC	Receivers of crystalloids only	Matched	73 (50 matched)	Scene (100%)	PHTRBC: Blunt: 93% Penetrating: 7% MVC 70% Fall from height 10% Matched control: Blunt: 94% Penetrating: 6% MVC 68% Fall from height 12%	PHTRBC: 34 (9‐75) Control: 35 (18‐75) *P* = .242
Price DD (1999)^34^	Oregon USA	1989‐1995	To evaluate the efficacy of early blood transfusion	Receivers of PHTRBC during air transport	Receivers of crystalloids in ground transport	Matched	84	n/d (100%)	n/d	n/d
Rehn M (2018)^35^	London UK	2009‐2015	To investigate the effect of PHTRBC on overall blood product use	“Code Red” patients after implementation of PHTRBC	“Code Red” patients before implementation of PHTRBC	Adjusted data	128	Scene (100%)	PHTRBC: Blunt: 64.8% Penetrating: 35.2% MVC: 42.2% Falls: 11.7% Control: Blunt: 68.6% Penetrating: 31.4% MVC: 42.3% Falls: 12.4% Other blunt: 13.9%	PHTRBC: 29 (25‐43) Control: 27 (19‐41)
Rehn M (2019)^36^	London UK	2009‐2015	To investigate the effect of PHTRBC on mortality	“Code Red” patients after implementation of PHTRBC	“Code Red” patients before implementation of PHTRBC	Adjusted data	239	Scene (100%)	PHTRBC: Blunt: 146 (61%) Penetrating 93 (39%) Control: Blunt: 189 (63%) Penetrating: 111 (37%)	n/d
Sumida MP (2000)^37^	Tennessee Connecticut USA	1995‐1996	To analyse the effect of PHTRBC on physiologic parameters and outcome	Receivers of PHTRBC	Receivers of crystalloids only	no	17	Scene+ interfacility (100%)	n/d	PHTRBC 28 Control: 27.8 *P* = .957
Prospective not‐comparative studies										
Chang R (2018)^25^	USA (9 trauma centers)	2015	To describe the phenotype and laboratory coagulation abnormalities of clinically evident coagulopathic bleeding (CC) after trauma	Highest‐risk trauma patients, CC+	CC‐	Adjusted data	PHTRBC in CC+ vs CC‐ 18 (44%) vs 82 (8%) *P* < .001	Scene (100%)	Overall: CC+ vs CC‐: Blunt: 28 (68%) vs 792 (81%) Penetrating: 12 (30%) vs 165 (17%) Both: 1 (2%) vs 21 (2%) Injury type *P* = .09	CC+:32 (25‐41) CC‐: 17 (8‐27) *P* < .001
Reed M (2017)^24^	Scotland	2013‐2015	To evaluate the prehospital activation of Code Red	Patients for whom a pre‐hospital Code Red was activated	None	n/a	16	n/d (100%)	Overall: Blunt: 44 (83%) Penetrating: 9 (17%)	Overall: 24 (14‐37)
Sherren PB (2013)^38^	Greater Sydney Area Australia	n/s (5 y)	To describe PHTRBC	Missions involving PHTRBC	None	n/a	147	n/d (100%)	Blunt: 93.9% Penetrating: 6.1% MVC: 79 Fall from height: 3.4% Other: 11.6%	RTS: 5.967 (4.083‐6.904)
Weaver AE (2012)^39^	London UK	2012	To examine the impact of on‐scene PHTRBC for seriously injured patients	Receivers of PHTRBC	None	n/a	50	Scene (100%)	n/d	n/d
Retrospective not‐comparative studies										
Berns KS (1998)^40^	Minnesota USA	1993‐1996	To document the development of protocols for and to review the experience with PHTRBC	Receivers of PHTRBC	None	n/a	94	scene+ interfacility (48%)	n/d	n/d
Bodnar D‐b (2014)^41^	Greater Brisbane Australia	2011‐2012	To describe the characteristics, clinical interventions and the outcomes of PHTRBC patients	Receivers of PHTRBC	None	n/a	71	Scene (100%)	Blunt: 73.2% Penetrating: 26.8% MVC 67%	Mean (SD) 32.1 (18.2)
Dalton AM (1993)^42^	Oregon Washington USA	1985‐1992	To show that PHTRBC is safe and practical	Receivers of PHTRBC with MAST	Receivers of PHTRBC without MAST	n/a	112	n/d (100%)	Overall: Blunt: 86% Penetrating: 14% MVC: 72%	Mean: MAST: 33 non‐MAST: 31
Fahy AS (2017)^43^	Minnesota USA	2002‐2014	To report our experience with a prehospital transfusion protocol in pediatric patients	Pediatric trauma patients receiving PHTRBC and/or ‐plasma	Pediatric non‐trauma patients receiving PHTRBC and/or ‐plasma	n/a	28^b^	scene+ interfacility (57%)	Blunt: 88% Penetrating: 12% MVC: 63% Gunshot wounds: 13%	24 (range 9‐66)
Heschl S (2018)^44^	Victoria Australia	2011‐2015	To describe the characteristics of PHTRBC	All cases where approval for PHTRBC was sought by paramedics	None	n/a	142	Scene (96%)	Blunt/penetrating: n/d MVC: 88% Crush/fall/other: 11.8%	mean (SD): 36.5 (15.8)
Higgins GL (2012)^45^	Maine USA	2007‐2008	To describe PHTRBC with respect to safety and efficacy and improvement in condition	receivers of PHTRBC	None	n/a	45	scene+ interfacility (71%)	n/d	n/d
Hooper N (2017)^46^	Southwest UK	2015‐2016	To describe experience with PHTRBC	Receivers of PHTRBC	None	n/a	62	n/d (84%)	n/d	n/d
Krook C (2018)^47^	Western Canada	2013‐2017	To describe the implementation and stewardship of a prehospital blood transfusion program	Receivers of PHTRBC	None	n/a	274	scene+ interfacility (74%)	n/d	n/d
Krugh D (1994)^48^	Ohio USA	1991‐1993	To describe and review the implementation of an off‐site blood product storage program	Receivers of PHTRBC	None	n/a	8	n/d (50%)	n/d	n/d
Lyon R (2017)^49^	Kent Surrey & Sussex UK	2013‐2014	To describe the characteristics of receivers of PHTRBC and evaluate their subsequent in‐hospital needs	Receivers of PHTRBC	None	n/a	147	scene (97%)	Blunt: 128 (87%) Penetrating: 14 (10%) MVC: 103 (73%) Fall from height: 17 (11.6%)	33 (SD 13.4)
Maher P (2017)^50^	Washington, USA	2015	To describe the development of a HEMS transfusion program	Receivers of PHTRBC or ‐plasma	None	n/a	RBC 13 FFP 3	scene+ interfacility (85%)	n/d	n/d
Mena‐Munoz J (2016)^51^	Pennsylvania Ohio Maryland USA	2003‐2012	To characterise receivers of out of hospital transfusion of blood products (mostly RBC and/or plasma) during critical care transport	Receivers of out of hospital blood products	None	n/a	1440^b^	Scene + interfacility (19%)	n/d	n/d
Mix FM (2018)^52^	Minnesota, USA	2011‐2015	To determine whether opportunities for blood product administration by ground ALS ambulances exist	Adult trauma patients with potential need for prehospital blood administration	None	n/a	28	Scene (100%)	Blunt: 26 (93%) Penetrating: 2 (7%)	n/d
Potter D (2015)^53^	Minnesota USA	2003‐2012	To summarize our initial experience with PHTRBC and PHT‐plasma in pediatric trauma patients	Receivers (<18 y) of PHTRBC and/or PHT‐plasma	None	n/a	16^b^	Scene + interfacility (100%)	Blunt: 87.5% Penetrating: 12.5%	Mean 30 (range 9‐66)
Raitt JE (2018)^54^	Thames Valley UK	2014‐2016	To review the appropriateness of PHTRBC and to identify outcomes	receivers of PHTRBC	None	n/a	n/a	Scene (95%)	Blunt: 53 (84%) Penetrating: 7 (11%) MVC 42 (67%) Fall 8 (13%)	ISS 34 (21‐43)
Sunde GA (2015)^55^	Bergen Norway	2014	To describe our preliminary results after implementing PHTRBC and PHT‐plasma	Receivers of PHTRBC and/or PHT‐plasma	None	n/a	4^b^	scene (75%)	Blunt: 67% Penetrating: 33%	n/d
Thiels CA (2016)^56^	Minnesota USA	2002‐2014	To report our experience with prehospital blood product transfusion	Non‐trauma patients receiving PHTRBC and/or ‐plasma	Trauma patients receiving PHTRBC and/or ‐plasma	no	PHTRBC 654	Scene + interfacility (36%)	n/d	n/d
Wheeler R (2013)^23^	New England USA	2005‐2009	To determine factors associated with hypothermia	trauma patients transported by HEMS, hypothermic on arrival	Non‐hypothermic trauma patients, transported by HEMS	n/a	30	Scene (100%)	n/d	(Mean ± SD): Hypothermic: 26.07 ± 11.86 Non‐hypoth: 15.53 ± 11.39
Case reports										
Garner AA (1999)^57^	Sydney Australia	1997	Case report			n/a	1^b^	Scene (100%)	Blunt: 100%	43 (*n* = 1)
Lawton LD (2012)^58^	Queensland Australia	n/s	Case report			n/a	1^b^	Scene (100%)	Blunt: 100%	n/d
Macnab AJ (1996)^59^	British Columbia Canada	1996	Case report			n/a	1	Interfacility (0%)	n/a	n/a
Description of protocol										
Trembley AL (2016)^60^	Minnesota Wisconsin USA	2016	Description of implementation of protocol	n/a	n/a	n/a	n/d	Scene + interfacility (n/d)	n/d	n/d
Vartanian, L (2017)^61^	Texas, USA	2016	Description of implementation of protocol	Receivers of PHTRBC and/or ‐plasma	None	n/a	12	n/d (67%)	Blunt: 7 (87%) Penetrating: 1 (12%) MVC: 5 (62%) Fall: 1 (8%)	n/d
**Military services**										
Prospective comparative studies										
Vitalis V (2017)^62^	French armed forces Sahel	2016‐2017	To evaluate the practices of battlefield transfusion (RBC, plasma, FWB)	Severely injured receivers of PHT‐RBC or ‐plasma or ‐FWB	No battlefield transfusion	No	7^b^ (4 of whom RBC)	POI + Role 1	Overall: Blunt: 1 (4%) Penetrating: 27 (96%) Explosion 16 (57%) Active external haemorrhage 12 (43%)	PHT: 45 (33‐52) Control: 25 (16‐22) *P* = .01
Retrospective comparative studies										
Howard, JT (2017)^63^	US military Afghanistan	2001‐2014	To evaluate potential influences on KIA mortality	Casualties who needed and received PHT	Casualties who needed but did not receive PHT	Adjusted data	75^c^	Prehospital helicopter transport to FST or CSH	Overall: Explosion: 65.1% Gunshot: 22.5% Blunt or other: 11.4%	n/d
O'Reilly DJ‐b (2014)^64^	UK MERT‐E Afghanistan	2006‐2011	To evaluate the effect of PHTRBC/PHT‐plasma on mortality	Receivers of PHTRBC and PHT‐plasma	Matched patients where no PHT available	Propensity score	97^b^	POI + Role 1	PHT: Blunt: 1% Penetrating: 99% Burn: 0% Explosive: 51.5% Gunshot wound: 47.4% Matched control: Blunt: 3.1% Penetrating: 96.9% Burn: 0% Explosive: 49.5% Gunshot wound: 47.4%	PHT: 16 (9‐25) Control: 16 (9‐24.5) *P* = .686
Shackelford S (2017)^65^	UK MERT, US Air Force Pedro, US DUSTOFF, Afghanistan	2012‐2015	To examine the association of PHTRBC and/or PHT‐plasma and time to initial transfusion with injury survival	receivers of PHTRBC and/or PHT‐plasma	no PHT	frequency matched	55^b^	POI to role 2 or 3	PHT: Explosives 84% Gunshot wound 16% ≥1 AMputation: 73% Hemorrhagic torso injury 56% Control: Explosives: 71% Gunshot wound: 29% *P* = .05 ≥1 AMputation: 27% *P* < .001 Hemorrhagic torso injury: 35% *P* = .004	PHT: 29 (17‐36) Control: 28.6 (24.0‐33.2) *P* = .88
Prospective not‐comparative studies										
Aye Maung N (2015)^66^	UK army Afghanistan	2012‐2014	To explore the utility and feasibility of forward transfusion of RBC	Missions where blood components were carried	None	n/a	2	POI + Role 1	Gunshot wound: 100% (*n* = 2)	n/d
Malsby RF (2013)^67^	US Army, Afghanistan	2012	Process improvement initiative of blood product transfusion on Urgent helicopter evacuation casualties	Receivers of PHTRBC and/or PHT‐plasma	None	n/a	15^b^	POI + Role 1	Explosion: 87% Gunshot wound: 13% ≥1 AMputation: 60%	n/d
Retrospective not‐comparative studies										
Barkana Y (1999)^68^	Israel Defense Force Medical Corps, Israel	1994‐1996	To characterise the different aspects of PHTRBC and to evaluate its potential effect on the morbidity and mortality	Receivers of PHTRBC	None	n/a	40	POI + Role 1	Blunt: 22.5% Penetrating: 77.5% Explosion: 47.5% Gunshot wounds: 22.5% Explosion + gunshot wounds: 7.5% MVC: 20% Fall from height: 2.5%	18 (11.5‐25)
Chen J (2017)^69^	Israeli Air Force, Israel	2003‐2010	To describe PHTRBC, and to evaluate adherence to clinical practice guidelines	Receivers of PHTRBC	None	n/a	89	Scene+ interfacility	Combat: 69% Non‐combat: 31% Gunshot wounds: 36% MVC: 28% Explosions: 24% Stab wound: 4% Plane crash: 2% Fall from height: 2%	n/d
Edgar IA (2014)^70^	US and UK military, Afghanistan	2011	To compare initial management and early outcomes in patients suffering bilateral lower limb amputations and differences related to the type of aeromedical evacuation assets	Surviving adult male patients with bilateral traumatic lower limb amputations transferred by MERT in a CH‐47 Chinook helicopter	vs transferred by PEDRO in an HH‐60 Pavehawk helicopter.	n/a	n/d	POI to Role 3	Only patients with bilateral lower limb amputations	NISS MERT: 27 (range 19‐41) PEDRO: 27 (range 22‐29) *P* = 1
Morrison JJ (2013)^71^	US and UK military Afghanistan	2008‐2011	To characterise and compare mortality among casualties evacuated with conventional military retrieval (CMR) to those evacuated with an advanced medical retrieval (AMR) capability	Casualties evacuated from POI by an AMR capability	Vs those evacuated by a medic‐led CMR capability	n/a	162^b^	POI to role 3	AMR: Blast: 70.4% Gunshot: 24.3% Other: 5.3% CMR: Blast: 60.8% *P* < .001 Gunshot: 34.9% Other: 4.3%	Mean (SD): AMR: 16 (17) CMR: 15 (16) *P* = .122
O'Reilly DJ‐a (2014)^72^	UK MERT‐E Afghanistan	2008‐2011	To present the initial experience of military PHTRBC and PHT‐plasma	Receivers of PHTRBC and/or PHT‐plasma	None	n/a	310^b^	POI to role 2 or 3	Blunt: 1.0% Penetrating: 99% Burn: 0.3% Explosive: 72.9% Gunshot wound 25.8%	20 (16‐29)
Powell‐Dunford N (2014)^73^	US Army, Afghanistan	2012	To enumerate the specific risks and risk management strategies of en route transfusion	Receivers of PHTRBC and/or PHT‐plasma	None	n/a	61^b^ (54 of whom RBC)	n/d	Explosion: 74% Gunshot wound 26%	n/d
Shlaifer A (2017)^74^	Israeli Defense Forces, Israel	2013‐2016	To describe feasibility, safety, adverse reactions, and adherence to clinical practice guidelines in PHT‐plasma	Receivers of PHT‐plasma. Among them 9 receivers of PHTRBC	None	n/a	9^b^	POI + Role 1	Penetrating: 68.5% Blunt: 15.2% Burn: 1.1% Blast: 1.1% Combination: 14.1%	ISS 1‐8:10.9% ISS 9‐14:20.7% ISS 16‐24:28.3% 25‐75:40.1%
Case reports										
West BC (2004)^75^	US Army Afghanistan	2004	Case report			n/a	1	POI to FST	Penetrating: 100% (*n* = 1)	n/d

*Note*: Median (IQR) unless otherwise specified.

Abbreviations: AMR, advanced medical retrieval; CC, clinically evident coagulopathic bleeding; CMR, conventional military retrieval; CSH, Combat Support Hospital; FFP, Fresh frozen plasma; FST, Forward Surgical Team; FWB, fresh whole blood; GI, gastro‐intestinal; HEMS, Helicopter Emergency Medical Service; ISS, Injury Severity Score; KIA, Killed in action; MAST, Medical antishock trousers; MERT(−E), Medical Emergency Response Team (−Enhanced); MVC, motor vehicle collision; n/a, not applicable; n/d, not described; n/s, not specified for PHTRBC; (N)ISS, (New) Injury Severity Score PHT, prehospital transfusion; PHTRBC, prehospital transfusion of red blood cells; PHT‐plasma, prehospital transfusion of plasma; POI, point of injury; RBC, red blood cells; RCT, randomised clinical trial; RTS, Revised trauma score; SD, SD; TIC, trauma induced coagulopathy; U, Units; UK, United Kingdom; USA, United States of America.

a PHTRBC unless otherwise specified; matched number of patients if applicable.

b PHTRBC and/or other prehospital blood component products.

c blood products not specified.

We included 14 articles discussing PHTRBC in military medical services, of which four studies had a control population. Four articles reported prehospital transfusion as an additional topic, while primarily discussing another aspect of their study^70^, ^71^, ^74^, ^76^ (Table 1).

No randomised trials were identified; all studies were observational. The potential overlap of patients in different manuscripts was substantial (Tables 2 and 3). The bias assessment of the studies is shown in Table 4.

**Table 2:** Included civilian studies by region and medical service, making potential overlapping study populations visible

Country	Region	First author (y)	Comments	85	86	87	88	89	90	91	92	93	94	95	96	97	98	99	00	01	02	03	04	05	06	07	08	09	10	11	12	13	14	15	16	17	18	19	20
Australia	(Greater) Brisbane	Bodnar D‐b (2014)^41^																																					
		Lawton LD (2012)^58^	Case report																																				
	(Greater) Sydney	Garner AA (1999)^57^	Case report																																				
		Sherren PB (2013)^38^	Exact period not specified																																				
	Victoria	Heschl S (2018)^44^																																					
Canada	Br. Columbia	Macnab AJ (1996)^59^	Case report																																				
	W. Canada	Krook C (2018)^47^	Shock Trauma Air Rescue Society (STARS)																																				
NL	Nijmegen, R'dam	Peters J (2017)^33^																																					
Norway	Bergen	Sunde GA (2015)^55^																																					
UK	Kent, Surrey & Sussex	Griggs JE (2018)^30^																																					
		Lyon R (2017)^49^																																					
	London	Rehn M (2018)^35^																																					
		Rehn M (2019)^36^																																					
		Weaver AE (2012)^39^																																					
	Scotland	Reed M (2017)^24^																																					
	South West	Hooper N (2017)^46^																																					
	Thames Valley	Raitt JE (2018)^54^																																					
USA	Alabama	Chang R (2018)^25^	9 trauma centers																																				
		Holcomb J (2017)^27^	9 trauma centers																																				
	Arizona	Chang R (2018)^25^	9 trauma centers																																				
		Holcomb J (2017)^27^	9 trauma centers																																				
		Brown JB‐a (2015)^28^	9 institutions																																				
	California	Chang R (2018)^25^	9 trauma centers																																				
		Holcomb J (2017)^27^	9 trauma centers																																				
	Connecticut	Sumida MP (2000)^37^																																					
	Illinois	Brown JB‐a (2015)^28^	9 institutions																																				
		Chang R (2018)^25^																																					
	Maryland	Holcomb J (2017)^27^	9 trauma centers																																				
		Mena‐Munoz J (2016)^51^																																					
		Berns KS (1998)^40^	Mayo HEMS																																				
		Brown JB‐a (2015)^28^	9 institutions; Trauma only																																				
		Chang R (2018)^25^	9 trauma centers																																				
		Fahy AS (2017)^43^	Mayo One, Mayo Pediatric/Neonatal Transport; Pediatric patients only																																				
		Holcomb J (2017)^27^	9 trauma centers																																				
	Minnesota	Kim BD (2012)^22^	Mayo HEMS; Trauma only																																				
		Mix FM (2018)^52^	Mayo HEMS and ground EMS; Trauma only																																				
		Parker ME (2017)^32^	Mayo HEMS; GI bleed only																																				
		Potter D (2015)^53^	Mayo HEMS; Pediatric patients only																																				
		Thiels CA (2016)^56^	Mayo HEMS																																				
		Trembley AL (2016)^60^	North Memorial Air Care; Description of protocol																																				
	New England	Brown JB‐a (2015)^28^ ^a^	9 institutions																																				
		Higgins GL (2012)^45^ ^b^																																					
		Wheeler R (2013)^23^ ^c^																																					
		Chang R (2018)^25^	9 trauma centers																																				
		Holcomb J (2017)^27^	9 trauma centers																																				
	Ohio	Krugh D (1994)^48^																																					
		Mena‐Munoz J (2016)^51^																																					
	Oregon	Chang R (2018)^25^	9 trauma centers																																				
		Dalton AM (1993)^42^																																					
		Holcomb J (2017)^27^	9 trauma centers																																				
		Price DD (1999)^34^																																					
	Pennsylvania	Brown JB‐a (2015)^28^	9 institutions																																				
		Brown JB‐b (2015)^29^	STAT MedEvac																																				
		Mena‐Munoz J (2016)^51^																																					
	Tennessee	Miller B (2016)^31^																																					
		Sumida MP (2000)^37^																																					
		Brown JB‐a (2015)^28^	9 institutions																																				
		Chang R (2018)^25^	9 trauma centers																																				
	Texas	Henriksen H (2016)^26^																																					
		Holcomb JB‐b (2015)^18^																																				
		Holcomb J (2017)^27^	9 trauma centers																																				
		Vartanian, L (2017)^61^	Cypress Creek EMS; Description of protocol																																				
		Brown JB‐a (2015)^28^	9 institutions																																				
		Chang R (2018)^25^	9 trauma centers																																				
	Washington	Dalton AM (1993)^42^																																					
		Holcomb J (2017)^27^	9 trauma centers																																				
		Maher P (2017)^50^																																					
	Wisconsin	Trembley AL (2016)^60^	North Memorial Air Care; Description of protocol																																				

Abbreviations: NL, the Netherlands; P.‐A.‐C. d'A., Provence‐Alpes‐Côte‐d'Azur; R'dam, Rotterdam.

a Massachusetts General Hospital.

b Lewiston and Bangor, LifeFlight of Maine.

c Dartmouth‐Hitchcock Medical Center, Lebanon, New Hampshire.

**Table 3:** Included military studies by region and medical service, making potential overlapping study populations visible

Subject	First author (y)	Comments	94	95	96	97	98	99	00	01	02	03	04	05	06	07	08	09	10	11	12	13	14	15	16	17
UK‐MERT Afghanistan	Aye Maung N (2015)^66^																									
	Edgar IA (2014)^70^																									
	Morrison JJ (2013)^71^																									
	O'Reilly DJ‐b (2014)^64^																									
	O'Reilly DJ‐a (2014)^72^																									
	Shackelford S (2017)^65^																									
USA Afghanistan	Edgar IA (2014)^70^	Pedro																								
	Howard, JT (2017)^63^	All helicopters																								
	Malsby RF (2013)^67^	Dustoff																								
	Morrison JJ (2013)^71^	Pedro/Dustoff																								
	Shackelford S (2017)^65^	Pedro/Dustoff																								
	Powell‐Dunford N (2014)^73^	Medevac helicopter																								
	West BC (2004)^75^	Case report																								
France Sahel	Vitalis V (2017)^62^																									
Israel	Barkana Y (1999)^68^																									
	Chen J (2017)^69^																									
	Shlaifer A (2017)^74^																									

Abbreviations: MERT, Medical Emergency Response Team; UK, United Kingdom; USA, United States of America.

**Table 4:** Risk of bias assessment, Newcastle‐Ottawa Scale

First author (y)	Selection	Comparability	Outcome
Civilian services			
Prospective comparative studies			
Henriksen H (2016)^26^	★★		★★
Holcomb J (2017)^27^	★★★	★★	★★
Retrospective comparative studies			
Brown JB‐a (2015)^28^	★★★	★★	★★
Brown JB‐b (2015)^29^	★★★★	★★	★★
Griggs JE (2018)^30^	★★★★	★★	★★★
Holcomb JB‐b (2015)^18^	★★★	★★	★★
Kim BD (2012)^22^	★★★		★★
Miller B (2016)^31^	★★	★★	★★
Parker ME (2017)^32^	★★★		★★
Peters J (2017)^33^	★★★		★★
Price DD (1999)^34^	★★	★	★★
Rehn M (2018)^35^	★★★★	★	★★
Rehn M (2019)^36^	★★★	★	★★★
Sumida MP (2000)^37^	★★★		★
Military services			
Prospective comparative studies			
Vitalis V (2017)^62^	★★★★		★★★
Retrospective comparative studies			
Howard, JT (2017)^63^	★★★	★	★★
O'Reilly DJ‐b (2014)^64^	★★★	★	★★
Shackelford S (2017)^65^	★★	★★	★★★

### 3.2 *Patient characteristics*

In total, 47 civilian studies reported on trauma patients. Blunt injury was most prevalent in the included studies, ranging from 55% to 100% of trauma patients.^18^, ^22^, ^24^, ^25^, ^26^, ^27^, ^28^, ^29^, ^30^, ^31^, ^33^, ^35^, ^36^, ^38^, ^41^, ^42^, ^43^, ^49^, ^52^, ^53^, ^54^, ^55^, ^57^, ^58^, ^61^ The mean or median injury severity score (ISS) varied from 18 to 43 (Table 1).^18^, ^22^, ^23^, ^24^, ^25^, ^26^, ^27^, ^28^, ^29^, ^30^, ^31^, ^33^, ^35^, ^37^, ^41^, ^42^, ^43^, ^44^, ^49^, ^53^, ^54^, ^57^ The most frequent mechanisms of injury were motor vehicle collisions (42%‐88%) 30, 33, 35, 38, 41, 42, 43, 44, 49, 54, 61 and falls from a height (3.4%‐13%).^30^, ^33^, ^35^, ^38^, ^49^, ^54^, ^61^

In military services, penetrating injuries were reported by 68% to 100% of studies 62, 64, 66, 68, 72, 74, 75, 76 and the mean or median ISS varied from 15 to 45 (Table 1).^62^, ^64^, ^65^, ^68^, ^70^, ^71^, ^72^ The most frequently reported mechanisms of injury were explosions (1%‐87%)^62^, ^64^, ^65^, ^67^, ^68^, ^69^, ^71^, ^72^, ^73^, ^74^, ^76^ and gunshot wounds (13%‐100%).^64^, ^65^, ^66^, ^67^, ^68^, ^69^, ^71^, ^72^, ^73^, ^76^

Data on non‐trauma patients were reported in 16 civilian studies and made up 3% to 100% of these study cohorts.^32^, ^40^, ^43^, ^44^, ^45^, ^46^, ^47^, ^48^, ^49^, ^50^, ^51^, ^54^, ^55^, ^56^, ^59^, ^61^ Suspected gastro‐intestinal bleeding or ruptured aortic aneurysm were the most often reported non‐traumatic diagnoses in transfused patients. Only one article reported on non‐trauma patients attended to by military services; the two patients were transferred between facilities and suffered from obstetric haemorrhage or respiratory disease, they accounted for 2% of transfused patients.^69^

### 3.3 *Outcome—Mortality*

#### 3.3.1 Civilian

Unadjusted data from one study suggested that less PHTRBC patients died compared with non‐receivers,^37^ while another unadjusted study found no difference.^32^

In propensity score‐matched trauma patients, Brown et al^28^, ^29^ found a significant advantage of PHTRBC on either 24 hours or 30 day mortality in two different studies, with 50 and 240 transfused patients, respectively. Rehn et al^36^ found a lower prehospital mortality in trauma patients transported from the scene. After adjustment, Holcomb et al^18^ found lower odds of mortality in critical trauma patients who received PHTRBC and/or plasma at 6 hours.

However, in six other studies reporting matched or adjusted data, no significant effect on mortality was found, either at 3 hours,^27^ 6 hours,^26^, ^30^ 24 hours,^26^, ^27^, ^31^, ^33^ 28 days^30^ or 30 days post‐infusion,^27^, ^33^ for in‐hospital mortality^26^, ^31^ or overall mortality^34^ (Table 5). Conversely, Kim et al^22^ found significantly lower mortality at 24 hours and a lower mortality overall for patients transfused with both PHT‐plasma and PHTRBC compared with patients receiving PHTRBC only. These studies varied in patient and injury characteristics, type of transport (from scene/ interfacility/ both), type of blood products used (RBC only or a combination of blood products), transfusion criteria as well as outcome measures, and therefore, data could not be meaningfully combined in a meta‐analysis.

**Table 5:** Outcomes

First author (y)	Mortality (*n* [%])	Shock on arrival to hospital^a^	24 h RBC requirement (U)	ICU/hospital LOS (d)	TIC^a^	Adverse events
Civilian services						
Prospective comparative studies						
Henriksen H (2016)^26^	PHT vs control					
	6 h: 10 (13.3%) vs 15 (8.3%) *P* = .210 24 h: 12 (16%) vs 19 (10.4%) *P* = .213 In‐hospital: 20(26.7%) vs 38(20.9%) *P* = .313	**SBP: 90 (77‐113) vs 100 (80‐125) *P* = .044** DBP: 59 (50‐69) vs 60 (48‐76) *P* = .299 HR: 111 (90‐133) vs 108 (85‐130) *P* = .425 **pH: 7.21 (7.06‐7.32) vs 7.27 (7.18‐7.33) *P* = .002** BE: −6 (−10 ‐ −3) vs −4 (−10 ‐ −1) *P* = .073	**10 (4‐15) vs 4 (2‐9) *P* < .001**	n/d	**rTEG MA: 62 vs 64 *P* = .02** **G‐value: 8.1 vs 8.69 *P* = .009** ACT: 121 vs 121 R‐time: 0.8 vs 0.8 K‐time: 1.65 vs 1.45 Angle: 70 vs 73 Ly30: 1 vs 1.4 Adjusting for PH‐RBC, PHT‐plasma associated with increased rTEG MA: **1 U increase in PH‐plasma was associated with (β coefficient) 13.95 mm (95%CI 3.13‐24.77 *P* = .012**	n/d
Henriksen H (2016)^26^	PHT vs control, matched					
	3 h: 4 (9.3%) vs 8 (12.1%) OR 0.74 (95% CI 0.24‐2.26) *P* = .60 24 h: 5 (11.6%) vs 10 (15.2%) OR 0.74 (95% CI 0.25‐2.17) *P* = .58 30d: 8 (18.6%) vs 14 (21.2%) OR 0.85 (95% CI 0.32‐2.28) *P* = .75	n/d	n/d	n/d	n/d	n/d
Retrospective comparative studies						
Brown JB‐a (2015)^28^	PHTRBC vs control, matched					
	**24 h: OR 0.02 (95% CI 0.01‐0.69) *P* = .04 30d: HR 0.12 (95% CI 0.03‐0.61) *P* = .01** *Scene patients*: 24 h: OR = 0.04 (95% CI 0.01‐1.12) *P* = .059 **30d: HR = 0.11 (95% CI 0.02‐0.54) *P* < .01**	**Admission hypotension: 60% vs 74% *P* = .02** BE −10 (−5 ‐ ‐12) vs −9 (−7 ‐ ‐12) *P* = .88	**14.0 (7.0‐21.7) vs 8.3 (3.4‐18.5) *P* = .03**	n/d	**I**NR > 1.5 OR = 0.01 (95% CI 0.01‐0.95) *P* = .05 Scene only: OR = 0.08 (95% CI 0.01‐1.35) *P* = .079	n/d
Brown JB‐b (2015)^29^	PHTRBC vs control, matched					
	24 h: 53 (22%) vs 86 (18%) *P* = .16 **In‐hospital: 74 (31%) vs 115 (24%) *P* = .03 24 h survival: AOR 4.91 (95% CI 1.51‐16.04) *P* = .01** In‐hospital survival: AOR 1.06 (95% CI 0.42‐2.61) *P* = .90 *Matched scene patients*: 24 h: 23 (32%) vs 37 (6%) *P* = .33 In‐hospital: 26 (37%) vs 48 (34%) *P* = .68 **24 h survival: AOR 6.31 (95% CI 1.88‐21.14) *P* < .01** In‐hospital survival: AOR 4.32 (95% CI 0.76‐24.72) *P* = .10	SBP 106 (80‐132) vs 110 (91‐130) *P* = .07 **“Shock on admission”; 139 (58%) vs 226 (47%) *P* < .01 AOR 0.28 (95% CI 0.09‐0.85) *P* = .03** *Matched scene patients*: **SBP 82 (60‐92) vs 104 (81‐126) *P* = .01** “Shock on admission”: 51 (72%) vs 99 (70%) *P* = .74 **AOR 0.24 (95% CI 0.07‐0.80) *P* = .02**	5 (2‐11) vs 4 (2‐9) *P* = .06 *Matched scene patients*: 8 (2‐18) vs 9 (3‐13) *P* = .66 **>4 U lower requirement; Coefficient − 4.5 U (95% CI ‐8.3 to −0.7) *P* = .02**	n/d	**INR: 1.4 (1.2‐1.9) vs 1.2 (1.1‐1.6) *P* < .01** **TIC: 113 (47%) vs 149 (31%) *P* < .01** AOR: 1.39 (95% CI 0.87‐2.24) *P* = .17 *Matched scene patients*: **INR 1.5 (1.2‐2.0) vs 1.3 (1.2‐1.6) *P* = .04** TIC: 37 (52%) vs 51 (36%) *P* = .06 AOR 2.02 (95% CI 0.53‐7.71) *P* = .30	ARDS: 5 (2%) vs 14 (3%) *P* = .61 *Matched scene patients*: ARDS: 3 (4%) vs 1 (1%) *P* = .07 PH transfusion reactions: none IH transfusion reactions: 1
Griggs JE (2018)^30^	PHTRBC vs control					
	6 h 10 (10%) vs 19 (18%) *P* = .2 AOR 0.48 (95% CI 0.19‐1.19) *P* = .11 28d: 21 (26%) vs 31 (40%), *P* = .09 AOR 0.66 (95% CI 0.32‐1.35) *P* = .26	n/d	3 (1‐8) vs 4.5 (2‐9) (no analysis) **≥4 units RBC in 24 h: 41 (40%) vs 62 (60%) *P* = .02** ≥10 units PRBC in 24 h: 14 (15%) vs 22 (22%) *P* = .31	n/d	n/d	No immediate transfusion complications
Holcomb JB‐b (2015)^18^	PHT vs control:					
	6 h: 12% vs 10% *P* = .425 Adjusted: OR 0.23 (95%CI 0.106‐1.056) *P* = .088 24 h: 14% vs 13% *P* = .529 Adjusted: OR 0.57 *P* = .176 30‐d: 22% vs 21% *P* = .626 Adjusted: OR 0.80 *P* = .478 **Adjusted, in critical patients: coefficient: 6 h: 0.23 (95% CI 0.062‐0.890) *P* = .033** 24 h: OR 0.57 *P* = .117 30d: OR 0.71 *P* = .441	SBP: 115 (90‐135) vs 112 (90‐138) *P* = .944 HR on arrival: 96 (78‐116) vs 98 (80‐116) *P* = .649 **BE: −3 (−6‐0) vs − 4 (−8 ‐ ‐1) *P* = .002** **pH: 7.30 (7.22‐7.35) vs 7.26 (7.19‐7.32) *P* = .003**	6 h RBC: 0 (0‐4) vs 1 (0‐5) *P* = .370 **Adjusted: Coefficient** **6 h transfusion: −3.72 (95% CI − 6.783 ‐ − 0.659) *P* = .017** **24 h transfusion: −3.64 − (95% CI 7.100 to −0.192) *P* = .039**	n/d	ACT: 113 (105‐128) vs 121 (105‐128) *P* = .546 α‐angle: 73 (69‐76) vs 72 (68‐76) *P* = .362 mA(mm): 64 (59‐68) vs 64 (59‐67) *P* = .270 LY30(%): 1.1 (0.2‐2.7) vs 1.3 (0.3‐2.9) *P* = .568	n/d
Kim BD (2012)^22^	PHT‐plasma+RBC vs PHTRBC:					
	6 h: 11% vs 4% *P* = .422 **24 h: 44% vs 10% *P* = .026** **Overall: 56% vs 18% *P* = .023**	SBP: 89 vs 109 *P* = .057 HR: 100 vs 109 *P* = .324 Lactate: 5.2 vs 4.4 *P* = .472 BE: −8.3 vs −8.4 *P* = .940 pH: 7.19 vs 7.22 *P* = .634	12.7 vs 11.4 *P* = .694	ICU: 6.3 vs 7.7 *P* = .672 Hospital: 11 vs 17 *P* = .352	**INR baseline: 2.6 vs 1.5 *P* = .004** **INR Arrival: 1.6 vs 1.3 *P* = .001** Change in INR^b^: 0.9 vs 0.2 *P* = .078 **Arrival aPTT: 51 vs 35 *P* = .037**	ARDS: 11% vs 8% *P* = .758 ARF: 0% vs 4% *P* = .600
Miller B (2016)^31^	PHTRBC vs control, matched:					
	24 h: 39 (20%) vs 31 (16%) *P* = .291 In‐hospital: 59 (30%) vs 48 (25%) *P* = .212 *Adjusted*: 24 h: OR 1.04 (95% CI 0.54‐1.98) *P* = .91 In‐hospital: OR 1.05 (95% CI 0.56‐1.96) *P* = .88	**SBP: 92.0 (77.2‐112.5) vs 110 (88.0‐124.0) *P* < .001** HR: 103 (84‐123) vs 106 (88‐122) *P* = .363	**6 (2‐12) vs 3 (0‐8) *P* < .001**	n/d	n/d	n/d
Parker ME (2017)^32^	PHT vs control:					
	30d: 13% vs 12% *P* = 1.00	*Pre vs post transport (mean SD)*: *PHT*: HCO3 (mmol/L): 23.20 ± 5.14 vs 22.41 ± 4.35 *P* = .27. Hemodynamic instability 55 (49%) vs 20 (18%) *P* < .001 *Control*: HCO3 (mmol/L) 23.67 ± 6.89 vs 21.90 ± 4.15 *P* = .29 Hemodynamic instability (%) 47 vs 18 *P* = .005	*PHT vs control*: Any RBC in‐hospital: 96 (86%) vs 40 (82%) *P* = .6 In‐hospital RBC: 4.0 (2.0‐6.0) vs 3.0 (2.0‐6.0) *P* = .84	Hospital: 5.0 (4.0‐8.0) vs 6.0 (4.0‐8.0) *P* = .52 ICU: 2.0 (1.0‐2.0) vs 2.0 (1.0‐3.0) *P* = .69	*Pre vs post transport (mean ± SD)*: *PHT*: INR 2.01 ± 1.51 vs 1.56 ± 0.83 *P* = .01 *Control*: INR 1.94 ± 0.97 vs 1.62 ± 1.37 *P* = .32	TRALI: 1
Peters J (2017)^33^	PHTRBC vs control, matched:					
	24 h: 19 (30%) vs 16 (32%) *P* = .531 30d: 22 (45%) vs 20 (40%) *P* = .547	BE: − 9.9 (−25.0 to −0.7) vs − 6.6 (−23.2 to −0.6) *P* = .628 Lactate (mmol/l): 3.6 (0.8‐21) vs 3.2 (1.1‐14.2) *P* = .142 “Shock on admission”: 26 (70%) vs 26 (58%) *P* = .243	**1443 mL (range: 0‐19 315 mL) vs 2240 mL (range: 0‐15 120 mL) *P* = .004** 24 h RBC including PH volume: 1958 mL (range: 270‐20 580) vs 2240 mL (range: 0‐15 120) *P* = .888	n/d	INR: 1.3 (range 1‐10) vs 1.3 (range 1‐3.1) *P* = .529 TIC: 14(40%) vs 10(26%) *P* = .188	PH transfusion reactions: none IH transfusion reactions: 1
Price DD (1999)^34^	PHTRBC vs control, matched:					
	Overall: 45% vs 40% *P* = .52	**HR: 113/min (SD 23) vs 98/min (SD 43) *P* = .002** SBP: n/sign **DBP: 69 mmHg (SD 19) vs 49 mmHg (SD 30) *P* = .003**	**In‐hospital RBC (mean (SD)): 1414 mL (SD 1660) vs 1007 mL (9351) *P* = .023**	ICU/hospital: n/sign	n/d	n/d
Rehn M (2018)^35^	PHTRBC vs control:					
	n/d	n/d	Total RBC (pre‐hospital + in‐hospital): 4 (2‐6) vs 6 (4‐12) **Univariate − 0.624 (95% CI −0.717 to −0.531) *P* < .001** **Multivariate – 0.671 (95% CI –0.767 to −0.574) *P* < .001**	n/d	n/d	PH/IH transfusion reactions: none
Rehn M (2019)^36^	PHTRBC vs control:					
	Overall: 143 (60%) vs 187 (62%) Univariate: OR 0.90 (95% CI 0.64‐1.28) *P* = .554 Multivariate: OR 0.92 (95% CI 0.64‐1.32) *P* = .648 **Prehospital: 66 (28%) vs 126 (42%)** ***P* < .001** **Univariate: OR 0.53 (0.36‐0.76) *P* < .001 Multivariate: OR 0.52 (95% CI 0.35‐0.78) *P* = .001**	n/d	0 (0‐5) vs 7 (4‐12)	n/d	n/d	n/d
Sumida MP (2000)^37^	PHTRBC vs control					
	**Overall mortality Frequency ratio: 1.2 vs 1.4 (Live‐1 Die‐2) *P* = .01**	Change in mean BP: 5.5 vs 15.6 *P* = .227 Change in mean HR: 7.6 vs −3.0 *P* = .159 **pH (mean): 7.23 vs 7.37 *P* = .008** **Bic (mean): 14.6 vs 21.4 *P* = .0001**	n/d	n/d	n/d	n/d
Prospective not‐comparative studies						
Chang R (2018)^25^	n/d for PHTRBC CC+ patients had increased mortality at all time points (all *P* < 0 .001)	n/d for PHTRBC	n/d for PHT	n/d for PHTRBC	CC+ vs CC‐: Received PHTRBC (n(%)): 18 (44%) vs 82 (8%) *P* < .001Transfused prehospital plasma: 18 (44%) vs 104 (11%) *P* < .001	n/d
Reed M (2017)^24^	n/d for PHTRBC	n/d for PHTRBC	n/d	n/d	**Coagulopathic patients received more blood component units prehospital (data not provided) *P* < .01**	n/d
Sherren PB (2013)^38^	Dead on scene: 22 (15%)	n/d	n/d	n/d	n/d	Transfusion reactions: none
Weaver AE (2012)^39^	60d: 52%	pH (mean): 7.07 BE (mean): −12.0	Mean: 10.5	n/d	n/d	n/d
Retrospective not‐comparative studies						
Berns KS (1998)^40^	Overall: 45% Trauma only: 52%	n/d	n/d	ICU (mean): 12 Hospital (mean): 22	n/d	Complications: none
Bodnar D‐b (2014)^41^	Trauma only: Dead on scene: 7 (9.9%) After arrival, in‐hospital: 25 (39%)	n/d	Mean (SD): 7.93 (7.18)	lCU: 5.5 (2,0‐16.25) Hospital: 15.0 (1 .0‐38.5)	n/d	n/d
Dalton AM (1993)^42^	24 h: 46 (41%) Overall: 51 (46%)	**Volume of blood and change in SBP: *P* = .20** MAST vs non‐MAST: SBP rise (mean): 38 mmHg vs 40 mmHg *P* = .29	n/d	n/d	n/d	PH transfusion reactions: 1 (DIB)
Fahy AS (2017)^43^	Trauma vs non‐trauma					
	30d: 2 (12%) vs 2 (17%) *P* = .39 Overall in‐hospital mortality: 14%	Lactate (mean ± SD): 2.4 ± 0.6 vs 3.2 ± 0.8 *P* = .09	**In‐hospital RBC (mean [range]): 4.3 (0‐8) vs 12.3 (0‐82) *P* = .03**	Hospital LOS (mean): 13.4 vs 8.9 *P* = .12	(mean (SD) INR 1.25 ± 0.4 vs 1.3 ± 0.3 *P* = .69 PTT: 29.9 ± 4.5 vs 31.5 ± 9.6 *P* = .58 TEG: K 2.8 ± 2.8 vs 2.9 ± 1.1 *P* = .94 Angle: 61 ± 15.3 vs 54.5 ± 9.2 *P* = .43 R: 4.9 ± 1.7 vs 9.6 ± 9.6 *P* = .04 MA: 55 ± 14.7 vs 59.8 ± 4.8 *P* = .55 Ly30: 0.75 ± 0.8 vs 0.03 ± 0.05 *P* = .08	Hemolysis: none Febrile non‐hemolytic reactions: none Anaphylaxis: none
Heschl S (2018)^44^	Trauma only: Dead on scene: 13 (9.6%) After arrival, in‐hospital: 36 (37.7%)	*Changes during treatment*: *arrival at scene ‐ start transfusion ‐ arrival hospital*: **HR: 116 (100‐130) to 119 (103‐132) to 112 (96‐130) *P* < .001** **SBP: 90 (80‐110) to 80 (65‐91) to 94 (71‐110) *P* < .001** **SI: 1.27 (1.00‐1.57) to 1.50 (1.20‐1.80) to 1.23 (0.98‐1.49) *P* = .004**	n/d	n/d	n/d	Complications: none
Higgins GL (2012)^45^	Prior to discharge: 31%	Pre‐ vs post‐transfusion: SBP < 90 mmHg: 71% vs 29% X2 = 9.29 df = 1 *P* = .002 MAP: 62 mmHg vs 82 mmHg t = −11.090 df = 3 *P* = .002	n/d	n/d	n/d	Transfusion reactions: none Complications: none
Hooper N (2017)^46^	Before arrival to hospital: 18%	n/d	n/d	n/d	n/d	n/d
Krook C (2018)^47^	Overall prehospital: 33 (12%)	n/d	n/d	n/d	n/d	adverse reactions: none
Krugh D (1994)^48^	5 (62.5%)	n/d	n/d	n/d	n/d	n/d
Lyon R (2017)^49^	Dead on scene: 38 (26%) After arrival to hospital: 6 h: 16% 28‐d: 30%	Mean (range) ± SD pH: 7.15 (6.60‐7.42) ± 0.17 BE(mEq/L): −9.48 (−28.20‐0.40) ± 6.82 Lactate(mmol/L): 5.27 (0.90‐19.90) ±4.08 **Changes during treatment: before transfusion vs at arrival to hospital:** **SBP increased *P* < .001** **DBP increased *P* < .001** **MAP increased *P* < .001** HR: *P* = .96 (data not provided)	n/d	ICU: 6 (2‐17) Hospital: 18 (3‐32)	n/d	Complications: none Ionized calcium (mean): 1.1 mmol/L; **Lower with increasing volume of PRBCs transfused *P* = .03**
Maher P (2017)^50^	PHTRBC: 5 (38%) PHT‐plasma: 1 (33%) Scene transports: 2 (22%) Interfacility transports: 4 (57%)	n/d	n/d	n/d	n/d	n/d
Mena‐Munoz J (2016)^51^	In‐hospital 30d: Overall: 22.5% (CI 20.4%‐25.0%) *PHTRBC vs no‐PHTRBC*: 201 (28%) vs 252 (27%) OR 0.77 (0.53‐1.13) **Transfused > 700 mL vs < 350 mL: 48 (47%) vs 161 (23%) OR = 2.11 (95% CI 1.21‐3.69)**	Overall: Lactate 2.4 (1.4‐4.8)	Odds of in‐hospital transfusion after PHTRBC: OR = 2.00 (95% CI 1.46‐2.76)	Overall: Hospital: 7 (3‐14) ICU: 4 (1‐9)	Overall: PTT: 32.1 (27.3‐38.6) INR: 1.4 (1.2‐1.8)	n/d
Mix FM (2018)^52^	n/d	n/d	n/d	n/d	n/d	n/d
Potter D (2015)^53^	Prior to discharge: 4 (25%)	Mean (range) Arrival Lactate: 3.6 mg/dL (1.1‐7.1) Arrival BE: −5.7 (−14.0 ‐ ‐4.0) Difference BE in‐transport vs arrival: unchanged in n = 3, improved in n = 2 (−8.0 to −6.0 after 3 U RBC and − 7.0 to −3.0 after 2 U RBC)	Mean 3.6 (range 0‐13)	Hospital: mean 9.3 (range 1‐45)	*Overall vs PHT‐plasma+RBC vs PHTRBC*: INR arrival (mean (range)): 1.4 (0.9‐2.7) vs 1.2 (0.9‐1.4) vs 1.5 (1.1‐2.7) (no analysis)	n/d
Raitt JE (2018)^54^	On scene: 9 (14%) In ED 8 (14%) In‐hospital 11 (19%) 15 (26.3%)	n/d	5 (range 1‐29)	n/d	n/d	n/d
Sunde GA (2015)^55^	On scene: 2 (50%) After arrival, prior to discharge: 0	n/d	n/d	n/d	n/d	Transfusion reactions: none Complications: none
Thiels CA (2016)^56^	*Overall*: 30d: 18.0% *Non‐trauma vs trauma*: **1d: 5% vs 10% *P* = .002** **30d: 16% vs 22% *P* = .03** *Surgical vs Medical vs GI‐bleed*: **1d: 6% vs 6% vs 2% *P* = .045** 30d: 15% vs 21% vs 13% *P* = .12	*Overall*: Hemodynamically unstable on admission: 124 (64%) *Non‐trauma vs trauma* **Lactate (mean ± SD): 3.2 ± 3.4 vs 3.3 ± 2.7 *P* = .003**	In‐hospital RBC (mean ± SD): *Non‐trauma vs trauma*: 7.1 ± 8.7 vs 8.2 ± 10.8 *P* = .19 *Surgical vs Medical vs G‐I bleed*: 7.4 ± 8.3 vs 8.2 ± 11.6 vs 6.1 ± 6.9 *P* = .51	Hospital LOS (mean ± SD): *Non‐trauma vs Trauma*: **9.4 ± 11.2 vs 12.2 ± 19.1 *P* = .009** *Surgical vs Medical vs GI‐bleed*: **12.1 ± 13.9 vs 9.5 ± 11.5 vs 6.3 ± 5.4 *P* < .001**	*Non‐trauma vs Trauma (mean ± SD)*: INR: 1.6 ± 0.8 vs 1.5 ± 1.0 *P* = .14 **PTT: 34.4 ± 13.5 vs 33.2 ± 13.7 *P* = .06** **TEG R‐time (min): 8.2 ± 6.6 vs 5.7 ± 3.1 *P* < .001** **A‐angle: 60.8 ± 15.1 vs 65.1 ± 9.7 *P* = .03** MA (mm): 60.0 ± 14.5 vs 61.9 ± 9.6 *P* = .61 LYS30(%): 2.2 ± 8.5 vs 1.4 ± 2.9 *P* = .19	Minor allergic reaction after additional in‐hospital plasma: 1 (0.1%) Volume overload: none TRALI: none Hemolytic transfusion reaction: none
Wheeler R (2013)^23^	n/d	n/d	n/d	Hypothermic vs non‐hypothermic (mean ± SD): ICU: 8.96 ± 8.72 vs 7.10 ± 8.51 Hospital: 18.20 ± 23.81 vs 8.67 ± 12.82	n/d	**PHTRBC vs controls: hypothermia OR 6.27 (95% CI 2.47‐14.89) *P* < .05** **PHTRBC in Oct‐Dec: OR 13.50 (85% CI 1.49‐165.25)**
Case reports						
Garner AA (1999)^57^	*n* = 1, survived	n/d	56 (*n* = 1)	ICU: 88 (*n* = 1)	n/d	n/d
Lawton LD (2012)^58^	*n* = 1, survived until ICU admission	n/d	n/d	n/d	n/d	n/d
Macnab AJ (1996)^59^	*n* = 1, survived until hospital admission	n/d	n/d	n/d	n/d	Hemolysis of donor red cell units during transit: 2 incidents because of improper packaging or cooling
Description of protocol						
Trembley AL (2016)^60^	n/d	n/d	n/d	n/d	n/d	Adverse effects: none
Vartanian, L (2017)^61^	Before hospital arrival: 1 (8%)	n/d	n/d	n/d	n/d	n/d
Military services						
Prospective comparative studies						
Vitalis V (2017)^62^	PHT vs control:					
	24 h: 2 (28.6%) vs 3 (14%) (no analysis performed)	n/d	Total in‐hospital RBC: 1 (0.25‐5.5) vs 0 (0‐2) *P* = .05	n/d	n/d	Complications: none
Retrospective comparative studies						
Howard, JT (2017)^63^	Needed & received PHT vs needed but no PHT:					
	KIA: AOR 0.17 (95% CI 0.06‐0.51, *P* = .002)	n/d	n/d	n/d	n/d	n/d
O'Reilly DJ‐b (2014)^64^	PHT vs control, matched:					
	**30d: 8 (8.2%) vs 19 (19.6%) *P* = .013**	SBP: 132 (111‐145) vs 131 (114‐150) *P* = .145 **HR: 92 (74‐115) vs 105 (82‐128) *P* = .041**	**In‐hospital RBC: 2 (1‐8.5) [0‐49] vs 0 (0‐3.5) [0‐26] *P* < .001** **Total RBC: 4 (2‐10) [0‐53] vs 0 (0‐3.5) [0‐26] *P* < .001**	n/d	n/d	n/d
Shackelford S (2017)^65^	PHT vs control, matched:					
	**24 h: 3 (5%) vs 69 (20%) (between‐group difference − 15% (95% CI − 22% ‐ − 7%) *P* = .007 AHR 0.26 (95% CI 0.08‐0.84) *P* = .02** **30d: 6 (11%) vs 78 (23%) (between‐group difference − 12% (95% CI − 21% ‐ − 2%) *P* = .05 AHR 0.39 (95% CI 0.16‐0.92) *P* = .03** 30d mortality in 24 h survivors: 3 (6%) vs 9 (3%) AHR 0.84 (95% CI 0.18‐4.00) *P* = .83 *Time to first transfusion and 24 h mortality*: **Time to transfusion < 15 min of MEDEVAC rescue (median 36 min after injury) vs delayed treatment: 2(3%) vs 68 (21%) AHR 0.17 (95% CI 0.04‐0.73) *P* = .02** **After sensitivity analysis allowing for transfusion futility: 2 (3%) vs 47 (16%) AHR 0.23 (95% CI 0.06‐0.96) *P* = .04** Time to transfusion 16 to 20 min after MEDEVAC vs delayed treatment: 10 (33%) vs 46 (17%) AHR 0.94 (95% CI 0.41‐2.17) *P* = .89	pH: 7.28 (7.17‐7.38) vs 7.29 (7.24‐7.34) *P* = .65 BE: −7 (−11 ‐ −4) vs −6.2 (−7.9 ‐ −4.4) *P* = .37 Shock on arrival: 42 (76%) vs 206 (69%) Adjusted for risk of prehospital death: AOR 1.01 (95% CI 0.86‐1.18) *P* = .94	**24 h RBC/WB: 15 (8‐23) vs 11 (8.5‐13.5) *P* = .002**	Hospital: 30 (21‐30) vs 30 (27‐33) *P* > .99	**INR: 1.40 (1.2‐1.7) vs 1.26 (1.16‐1.36) *P* = .008**	n/d
Prospective not‐comparative studies						
Aye Maung N (2015)^66^	Overall: 0% (*n* = 2)	Changes during treatment: radial pulse returned (*n* = 2)	n/d	n/d	n/d	Adverse events: none Out‐of‐standard blood product temperature: 7 incidents
Malsby RF (2013)^67^	24 h: 2 (33%, *n* = 6))	BE (*n* = 5): −7 (−7 ‐ ‐4) Pre‐ vs post‐transfusion: SBP 99 (80‐116) vs 120 (104‐134) HR 132 (128‐138) vs 123 (112‐138)	10 (3.5‐14.5) (*n* = 7)	n/d	n/d	Adverse reactions: none Out‐of‐standard blood product temperature: none
Retrospective not‐comparative studies						
Barkana Y (1999)^68^	In‐hospital: 16%	SBP on arrival: 110	“Emergency phase RBC”: 5 (0‐4)	n/d	n/d	Adverse reaction: 1 (rash)
Chen J (2017)^69^	Overall: 10 (11%) On arrival to hospital: 7 (8%) 24 h: 9 (10%) In‐hospital: 3 (3%)	Scene vs hospital arrival: SBP: 119 (90‐130) vs 120 (80‐130) *P* = .49 DBP: 70 (60‐80) vs 70 (60‐80)*P* = .23 **HR: 119 (100‐130) vs 108 (90‐120) *P* < .01** SI: 1 (0.78‐1.24) vs 0.94 (0.73‐1.5) *P* = .89	n/d	n/d	n/d	Adverse reactions: none Immediate transfusion‐related complications: none Technical problems: none
Edgar IA (2014)^70^	After arrival, in‐hospital: 4.5% (n/s for PHTRBC)	MERT vs PEDRO: SBP: 130 (61‐170) vs 75 (46‐108) *P* = .0849 HR: 112 (80‐152) vs 89 (62‐150) *P* = .3629 pH: 7.26 (6.9‐7.5) vs 7.27 (7.22‐7.32) (no analysis) **Increased volumes of PHT and SBP closer to physiological normal at arrival to role 3 *P* = .0296 (data not provided)**	MERT vs PEDRO: RBC in ED: 5 (2‐14) vs 12 (6‐21) (no analysis) **Correlation between use of increased volumes of PHT and reduced total transfusion requirement *P* < .0194 (data not provided**)	n/d	n/d	n/d
Morrison JJ (2013)^71^	*AMR vs CMR*: overall: 9.1% vs 9.2% *P* = .536 (n/s for PHTRBC)	n/d	n/d	n/d	n/d	n/d
O'Reilly DJ‐a (2014)^72^	Overall: 62 (20%)	n/d	7 (1‐15) total RBC: 8 (3‐18)	n/d	n/d	Adverse effects: none Complications: none
Powell‐Dunford N (2014)^73^	24 h: 8 (13%)	BE: −9 (−14 ‐ −6) *Pre‐ vs post‐transfusion*: SBP: 86 (70‐104) vs 108 (85‐127) *P* = .001 DBP: 52 (40‐66) vs 60 (47‐71) HR: 133 (125‐141) vs 125 (110‐138) *P* = .000 SI: 1.6 (1.2‐2.0) vs 1.1 (1.0‐1.5) *P* < .01 MSI: 2.2 (1.7‐2.6) vs 1.7 (1.3‐2.1) *P* = .000	10 (8‐14)	n/d	INR: 1.2 (1.1‐1.4)	Adverse reaction: none
Shlaifer A (2017)^74^	In‐hospital: 11 (12%) (n/s for PHTRBC)	n/d	n/d	ICU: 1‐3d: 19(35%) 4‐6d: 12(22%) 7‐13:13(24%) 14d+: 10(19%) Hospital: ≤6d: 24(26%) 7‐13d: 24(26%) 14‐20d: 10(11%) 21d+: 34(37%) (n/s for PHTRBC)	n/d	Adverse event to FDP: 1 (chills and shivering)
Case reports						
West BC (2004)^75^	*n* = 1, survived to facility	SBP improved from unobtainable to 100 (*n* = 1)	n/d	n/d	n/d	Adverse reactions: none

In bold values represents statistically significant results.

*Note*: Values presented as median (IQR) unless otherwise specified. Available *P*‐values have been presented. Boldface indicates significant outcomes.

Abbreviations: ACT, activated clotting time; (A)HR: (Adjusted) Hazard Ratio AMR: Advanced Medical Retrieval (A)OR: (Adjusted) Odds Ratio aPTT, activated partial thromboplastin time; ARDS, Acute Respiratory Distress Syndrome ARF, acute renal failure BE, Base excess Bic, bicarbonate; CC, clinically evident coagulopathic bleeding; CI, Confidence Interval CMR, Conventional Military Retrieval DBP, diastolic blood pressure DIB, difficulty in breathing DOW, Died of Wounds (died after arrival to facility) (F)WB, (Fresh) Whole Blood GI, gastro‐intestinal HR, Heart Rate ICU, intensive care unit IH, in‐hospital INR, International Normalized Ratio IQR, Interquartile Range KIA, Killed in action (died before arrival to facility) LOS, length of stay MAP, Mean arterial pressure MAST, medical antishock trousers MERT, Medical Emergency Response Team MSI, Modified shock index (heart rate/mean blood pressure); n/d, not described n/s, not specified n/sign, not significant PH, prehospital PHT, prehospital transfusion PHT‐plasma, prehospital transfusion of plasma; PHTRBC, Prehospital Transfusion of Red Blood Cells RBC, Red blood cells SBP, Systolic Blood Pressure SI, Shock Index TEG, Thromboelastography TIC, trauma induced coagulopathy TRALI, Transfusion related lung injury; U, units.

a Values at arrival to hospital, unless otherwise specified.

b A positive INR Change denotes an improvement in coagulopathy.

#### 3.3.2 Military

In military services, almost all studies included patients who possibly received other prehospital blood products besides RBC. Two retrospective studies compared trauma PHT recipients to non‐receivers, and found significantly lower mortality in PHT patients (either overall, 24 hours or 30‐day mortality) (Table 5).^65^, ^72^ One of these studies subsequently focused on those patients who survived the first 24 hours; the beneficial effect on 30‐day mortality was no longer present.^65^ This concurs with a large retrospective study (with a partially overlapping study population), where the odds for “killed in action” (KIA) mortality (death before arrival at treatment facility) was 83% lower for casualties who needed and received prehospital transfusion, compared with patients who needed but did not receive a prehospital transfusion.^63^

### 3.4 *Outcome—Shock after transfusion*

#### 3.4.1 Civilian

Six observational studies compared vital parameters or POCT results before and after transfusion.^32^, ^42^, ^44^, ^45^, ^49^, ^53^ Five of these noted significant beneficial effects of PHTRBC (decrease in heart rate [HR] and shock index [SI],^44^ rise in systolic,^44^, ^49^ diastolic^49^ or mean arterial blood pressures,^45^, ^49^ less hypotensive episodes (ie, SBP < 90 mmHg)^45^ or less “haemodynamic instability”^32^) (Table 5).

Studies comparing vital parameters in PHTRBC patients vs non‐receivers report conflicting results: significantly lower occurrence of hypotension,^28^ a higher DBP,^34^ and a higher BE and pH^18^ have been reported in PHTRBC patients, but in contrast, significantly lower SBP,^26^, ^29^, ^31^ a higher HR,^34^ a lower pH^26^, ^37^ and a lower bicarbonate level^37^ have also been found. Other studies found no significant differences in either SBP,^18^, ^34^ DBP,^26^ HR,^18^, ^26^, ^31^ BE,^26^, ^28^, ^33^ lactate,^33^ change in mean BP or in HR^37^ or occurrence of “shock on admission.”^33^ Kim et al^22^ compared PHTRBC with PHTRBC + PHT‐plasma and found no significant differences in SBP, HR, lactate, BE or pH. Brown et al^29^ measured base deficit and lactate levels on arrival to hospital, and used these to calculate the odds of shock. They found that in PHTRBC patients, these odds were significantly lower than in matched control patients (Table 5).

#### 3.4.2 Military

In military EMS, three observational studies analysed the change in vital parameters after transfusion, all showing improvements (significant rise in SBP and improvement in SI^73^; a fall in HR^69^, ^73^; or a SBP closer to physiologically normal values as the prehospital transfused volume increased^70^).

Only one study comparing PHTRBC patients to controls found a significant difference reporting a lower HR.^72^ No significant differences were found for SBP, pH, BE, or “shock on arrival”^65^, ^72^ (Table 5).

### 3.5 *Outcome—24‐hour RBC Requirement*

#### 3.5.1 Civilian

In the first 24 hours after admission to hospital, civilian patients received a median of 0 to 14 U of RBC,^22^, ^26^, ^28^, ^29^, ^30^, ^31^, ^33^, ^36^, ^39^, ^41^, ^54^ and paediatric patients received a mean of 3.6 U.^53^

Five analyses found the RBC requirement in‐hospital or in the first 24 hours to be significantly higher for PHTRBC patients,^26^, ^28^, ^31^, ^34^, ^43^ whereas five others found it to be significantly lower.^18^, ^29^, ^30^, ^33^, ^35^ One of these studies noted that taking the prehospital transfused volume into account, the cumulative 24 hours RBC requirement was not significantly different.^33^ Two other studies found no significant difference in in‐hospital or 24 hours RBC requirement in either PHT vs control or in PHTRBC vs PHTRBC+PHT plasma^22^, ^32^ (Table 5).

#### 3.5.2 Military

Median RBC requirement in the first 24 hours after arrival to hospital was 5 to 10 units,^67^, ^68^, ^73^ one study reporting a median of 15 units of RBC/fresh whole blood in 24 hours.^65^ One study focusing on double amputees showed that an increased volume of prehospital transfused blood was significantly associated with a decreased transfusion requirement in the emergency department.^70^ However, three comparative studies observed (an almost) significantly higher 24 hours or in‐hospital transfusion requirement in PHTRBC patients.^62^, ^65^, ^72^

### 3.6 *Outcome—Signs of trauma‐induced coagulopathy (TIC) on arrival to hospital*

Three civilian studies compared the international normalised ratio (INR) of patients who received PHTRBC vs patients who did not. One study reported that PHTRBC patients had significantly lower odds of TIC 28 on arrival to hospital, while two other studies did not find an association between PHTRBC and INR.^29^, ^33^ One study reported that coagulopathic patients had received significantly more units of PHTRBC than non‐coagulopathic patients.^24^ All the other studies reporting on coagulation state are biased by the use of prehospital plasma besides the PHTRBC^18^, ^22^, ^25^, ^26^, ^32^, ^43^, ^56^, ^65^ (Table 5).

### 3.7 *Outcome—Length of stay in ICU/in hospital*

Three civilian studies compared length of stay (LOS) in ICU and LOS in hospitals for PHTRBC patients and their matched controls. No significant differences were found.^22^, ^32^, ^34^

Only one military study describes LOS, finding a median hospital LOS of 30 days for both PHTRBC patients and controls.^65^

### 3.8 *Outcome—Safety/adverse events*

Most studies on civilian EMS (11) reported no transfusion reactions occurring.^30^, ^35^, ^38^, ^40^, ^43^, ^44^, ^45^, ^47^, ^49^, ^55^, ^60^ A lung injury associated with a transfusion was reported,^32^ and there was one possible adverse reaction in a trauma patient who developed shortness of breath, which was interpreted as secondary to volume overload.^42^ A case report has described two “near miss” incidents where haemolysis of donor cells occurred during transport, when the units had not been packed correctly.^59^

Patients transfused before arrival to hospital were more likely to be hypothermic^23^ and have lower calcium levels,^49^ but there was no significant difference in the occurrence of acute respiratory distress syndrome (ARDS) in PHTRBC, non‐PHTRBC and PHTRBC+PHT‐plasma patients.^22^, ^29^

As in civilian services, seven military studies have reported no adverse reactions to PHTRBC.^62^, ^64^, ^66^, ^67^, ^69^, ^74^, ^75^ One possible transfusion reaction is described, in which a patient developed a fine rash on their trunk after one unit of RBC.^68^ Seven incidents were reported where the blood products were found to have an out‐of‐standard temperature.^66^

## 4 DISCUSSION

This systematic review summarises the literature regarding the effects of PHTRBC on several outcome measures.

Overall, evidence of an effect of PHTRBC on outcomes is of limited quality. Notably, no controlled studies were identified, and all studies were observational. Therefore, all reported treatment effects must be interpreted with care. Confounding is likely—and residual confounding cannot be excluded in those studies that attempted to control for confounding—such that causal inferences on the effect of PHTRBC on outcomes are essentially not possible.^77^ Nonetheless, in the absence of controlled trials, these studies represent the best available evidence and may at least provide some insight about possible associations between PHTRBC and outcomes.

The high heterogeneity of the studies was a second factor, which impedes the interpretation of the reported data. As we had expected, patients transported by civilian and military services differed considerably with respect to injury type, injury severity and mortality rates. However, also within these groups, heterogeneity in injury type, injury severity, type of transport, transfusion criteria and type of intervention prevented meaningful meta‐analysis. Differences between study outcomes might at least in part be explained by these factors.

### 4.1 *Mortality*

Results on overall mortality are conflicting, and we found no consistent evidence for any effects of PHTRBC on survival. Recently, Rijnhout et al^78^ published a meta‐analysis on the effects of prehospital transfusion on mortality. In line with our results, these authors did not observe an effect of PHTRBC (without simultaneous transfusion of plasma) on mortality. For the 24 hours mortality, an odds ratio of 0.92 was reported, with a broad 95% confidence interval (0.46‐1.85) that does not exclude clinically important effects of PHTRBC in either direction, which indicates an inconclusive result.^79^ Importantly, heterogeneity was high (I^2^ 80%), similar to the heterogeneity that we observed in explorative analyses. While quantitative heterogeneity was lower for long‐term survival, qualitative heterogeneity, along with limited quality and a substantial potential for residual confounding in the observational studies, prompted us to question whether it was appropriate to report a pooled effect estimate. Nonetheless, despite these limitations, the data by Rijnhout et al can be considered hypothesis generating and do suggest that the combination of PHTRBC and plasma may potentially be beneficial for long‐term survival, warranting further investigation. Similarly, a recent systematic review by Shand et al reported high heterogeneity and the authors could not draw conclusions about the effect of prehospital transfusion of any blood component on outcome.^80^ Previous systematic reviews have summarised the evidence up to 2015^81^ and 2016,^82^ however, numerous studies have been published thereafter such that a more up to date systematic review is warranted.

### 4.2 *Haemodynamics, coagulopathy, 24‐hours RBC requirement and LOH/ICU stay*

Observational studies in both civilian and military services found that after PHTRBC, SBP recovers, HR decreases and SI improves. However, these improvements could be due to the administration of analgesia or fluids in general or merely be time‐dependent effects. Outcomes of comparative studies in both military and civilian services reporting on haemodynamics, coagulopathy or 24‐hour RBC requirement are conflicting and could not confirm an effect of PHTRBC on any of these variables.

A large majority of patients in both civilian and military observational studies required transfusion after arrival to hospital, which may be seen as confirmation of the appropriateness of prehospital transfusions. Some studies reported a higher 24 hours RBC requirement in PHTRBC patients, while others reported this to be lower. A higher 24 hours RBC requirement may suggest that the patients who were bleeding most severely had been identified correctly in the prehospital setting as requiring PHTRBC. These patients, in turn, also have a higher demand for blood products when at the hospital. An explanation of a lower 24 hours RBC requirement in PHTRBC patients could be that these patients bled less through prevention of coagulopathy and thus required less transfusion. However, there is thus far no evidence that PHTRBC generally reduces the occurrence of TIC. In some cases, the PHTRBC patients may merely have received the blood they needed earlier, resulting in a lower 24 hours in‐hospital RBC requirement. There is currently no evidence that PHTRBC has influence on LOS in hospital and LOS in ICU.

### 4.3 *Adverse events*

There have been few instances of transfusion reactions being reported after PHTRBC. Fortunately, transfusion reactions in the general population are rare, with urticaria occurring in 1% to 3% of patients, febrile non‐haemolytic transfusion reaction and cardiac overload in <1%, and all other transfusion reactions in <0.1%.^83^

### 4.4 *Strength and limitations*

We performed a thorough search with broad inclusion criteria. The eligibility of studies was assessed independently by two authors. It is therefore unlikely that we have overlooked data that would significantly alter our conclusions. Additionally, despite a different patient population, mechanism of injury and medical facilities, we did not exclude military services but decided to report on them separately, thereby including an important source of data on PHTRBC.

No controlled trials were identified, most observational studies were retrospective, and high heterogeneity also precluded meaningful data pooling. While this at first glance seems a limitation of this systematic review, it is rather a limitation of previous research and in fact an important outcome of this study; this finding highlights the lack of high‐quality outcome studies, and suggests that randomised trials are needed for more conclusive evidence on the causal relationship between PHTRBC and patient outcomes.

## 5 CONCLUSION

This systematic review revealed that despite increasing use of PHTRBC by civilian EMS, high‐quality evidence for beneficial effects is still lacking. In the absence of high‐quality data, it seems reasonable to assume that massively bleeding patients may benefit from PHTRBC. This assumption is supported by several observational studies that do suggest possible beneficial effects on mortality. This may especially be true when PHTRBC is combined with plasma administration. PHTRBC also appears to improve haemodynamic parameters, but there is no evidence that shock on arrival to hospital is averted, nor of an association with TIC or LOS in either hospitals or ICUs. Few adverse events have been reported. Given that prevention is generally better than treatment, prevention of haemorrhagic shock through compression of external bleeding, stabilisation of pelvic fractures, prevention of hypothermia and the administration of tranexamic acid should still remain a priority in trauma patients, even when PHTRBC is available.

## CONFLICT OF INTEREST

The authors declare no conflicts of interest.
